# Orientated Immobilization of FAD-Dependent Glucose Dehydrogenase on Electrode by Carbohydrate-Binding Module Fusion for Efficient Glucose Assay

**DOI:** 10.3390/ijms22115529

**Published:** 2021-05-24

**Authors:** Qingye Han, Weili Gong, Zhenyu Zhang, Lushan Wang, Binglian Wang, Lei Cai, Qingjun Meng, Yiwei Li, Qingai Liu, Yan Yang, Lan Zheng, Yaohong Ma

**Affiliations:** 1 Shandong Provincial Key Laboratory of Biosensors, Biology Institute, Qilu University of Technology (Shandong Academy of Sciences), Jinan 250103, China; h18651730030@163.com (Q.H.); zhangzhenyu1113@163.com (Z.Z.); wangbinglian79@126.com (B.W.); cailei@sdas.org (L.C.); mengqjun2008@126.com (Q.M.); mr.know-nothing@163.com (Y.L.); liuqingai1986@163.com (Q.L.); ihatee@126.com (Y.Y.); zhlan8409@163.com (L.Z.); 2 State Key Laboratory of Microbial Technology, Institute of Microbial Technology, Shandong University, Qingdao 266237, China; lswang@sdu.edu.cn

**Keywords:** FAD-dependent glucose 1-dehydrogenase, nanocellulose, carbohydrate-binding module family 2 tag, orientated immobilization, biosensor

## Abstract

The discovery or engineering of fungus-derived FAD-dependent glucose 1-dehydrogenase (FAD-GDH) is especially important in the fabrication and performance of glucose biosensors. In this study, a novel FAD-GDH gene, phylogenetically distantly with other FAD-GDHs from *Aspergillus* species, was identified. Additionally, the wild-type GDH enzyme, and its fusion enzyme (GDH-NL-CBM2) with a carbohydrate binding module family 2 (CBM2) tag attached by a natural linker (NL), were successfully heterogeneously expressed. In addition, while the GDH was randomly immobilized on the electrode by conventional methods, the GDH-NL-CBM2 was orientationally immobilized on the nanocellulose-modified electrode by the CBM2 affinity adsorption tag through a simple one-step approach. A comparison of the performance of the two electrodes demonstrated that both electrodes responded linearly to glucose in the range of 0.12 to 40.7 mM with a coefficient of determination R^2^ > 0.999, but the sensitivity of immobilized GDH-NL-CBM2 (2.1362 × 10^−^^2^ A/(M*cm^2^)) was about 1-fold higher than that of GDH (1.2067 × 10^−2^ A/(M*cm^2^)). Moreover, a lower detection limit (51 µM), better reproducibility (<5%) and stability, and shorter response time (≈18 s) and activation time were observed for the GDH-NL-CBM2-modified electrode. This facile and easy immobilization approach used in the preparation of a GDH biosensor may open up new avenues in the development of high-performance amperometric biosensors.

## 1. Introduction

As an important substance in the management of diabetes and control of food quality and the fermentation process, glucose levels need to be frequently monitored [1]. Among currently available glucose monitoring methods, enzyme-based electrochemical glucose biosensors with various advantages, such as being simple in operation, quick in measurement and accurate in results, have attracted extensive attention in the field of glucose monitoring. Various oxidoreductases have been used in glucose biosensors. In particular, glucose oxidase (GOx), which utilizes O_2_ as the natural electron acceptor and simultaneously produces H_2_O_2_, has been the most widely used oxidoreductase [2]. However, the reactions catalyzed by GOx are easily affected by the dissolved O_2_ concentration; if the detection is based on measuring the H_2_O_2_ level, the electrode surface reactions are affected by the applied, extreme potential (usually over +600 mV vs. standard electrode), which opens up the sensor system for interfering reactions and causes significant bias in the measurement [3]. To circumvent this problem, alternative enzymes, especially glucose dehydrogenase (GDH), which are insensitive to oxygen and use artificial redox mediators with a lower potential range to shuttle electrons from the enzyme to the electrode, have become attractive for biosensors [4].

According to their cofactors and origin, GDHs can be grouped into different types. Among them, fungus-derived GDHs (EC 1.1.5.9, fFAD-GDH) with tightly bound flavin adenine dinucleotide (FAD) as cofactors not only are insensitive to O_2_ but also display high substrate specificity to glucose and thus are especially attractive enzymes for use in glucose biosensor applications [5,6,7]. To date, several fFAD-GDHs have been reported, but as an enzyme group that has just recently started to attract significant attention, the discovery or engineering of novel FAD-GDHs with practical properties for glucose sensing applications becomes particularly attractive [7]. Recently, developed glucose sensors using fFAD-GDHs are mainly of the mediator type, in which the analyte (glucose) is oxidized by immobilized fFAD-GDH; then, the cofactor FAD is reduced to FADH2, followed by the FADH2-mediated reduction of an artificial electron acceptor (mediator), which is then re-oxidized at the electrode to generate a response current [8]. For more effective application of this biosensor, apart from high substrate selectivity, the bioelectrochemical devices in these fields of application are expected to demonstrate distinct performances, including high current output, high sensitivity, short response time, high reproducibility, and high stability [7]. Thus, the structure and function of immobilized fFAD-GDHs have to be maintained to preserve their biological activity after immobilization, and they are expected to remain tightly bound to the electrode surface and not to be desorbed during the use of the biosensor.

As comprehensively reviewed in previous studies, a wide range of enzyme immobilization strategies, including physical adsorption, covalent cross-linking, entrapment, and affinity, have been developed [9,10]. However, the former three strategies conducted by using randomly distributed active groups usually result in non-oriented enzyme immobilization, which further brings about structural deformation and the shielding of active binding sites of the enzymes. In contrast, the latter method, based on forming affinity bonds between a support and a specific tag fused to the enzyme, which allows the control of the orientation of the biomolecule in order to facilitate the efficient diffusion of the substrate and mediator through the enyzme internal cavity toward the catalytic site, as well as interfacial electron transfer between the mediator and electrode, exhibits promising potential in the development of biosensors with the expected performances [9,11,12].

Recently, various affinity tags, such as histidine (His), cysteine (Cys), biotin acceptor peptide, or carbohydrate-binding modules (CBMs), were reported and fused to the amino or carboxyl terminal of the target enzymes at the gene level, which confer novel affinity features to different supports (e.g., gold, crystalline nanocellulose) [13]. Among the tags, cellulose-binding CBMs, as a kind of affinity tag for the purification and immobilization of proteins, exhibit great advantages for biosensor applications [14]. It has been reported that certain properties, such as the enzymatic efficiency and stability, of some industrially important enzymes were significantly enhanced by their fusion with CBMs and immobilization on cellulose [15,16,17]. In addition, the interaction of some CBMs from families 2 and 3 with cellulose has been characterized as “irreversible”, requiring strong denaturing conditions to desorb [18], which cannot be easily broken when the condition changes during analysis, thus guaranteeing good stability and reproducibility. Furthermore, cellulose with a series of desirable inherent characteristics, such as inertness, biocompatibility, low non-specific protein binding, disposability, affordability, and safety, has been considered as an ideal support for enzyme immobilization for various applications [19].

In this study, after identifying a novel FAD-GDH gene from *Aspergillus niger* (*A. niger*) An76, we heterologously expressed the wild-type FAD-GDH and a fusion FAD-GDH with CBM tag from family 2 in *Pichia pastoris*. Then, they were immobilized on electrodes either by glutaraldehyde cross-linking or affinity adsorption. The detection performance, including linear range, detection limit, sensitivity, substrate specificity, repeatability, stability, and anti-interference capability of both FAD-GDH sensing elements was analytically studied and compared.

## 2. Results and Discussion

### 2.1. Heterologous Expression and Purification of GDH and GDH-NL-CBM2

**Figure 1:**
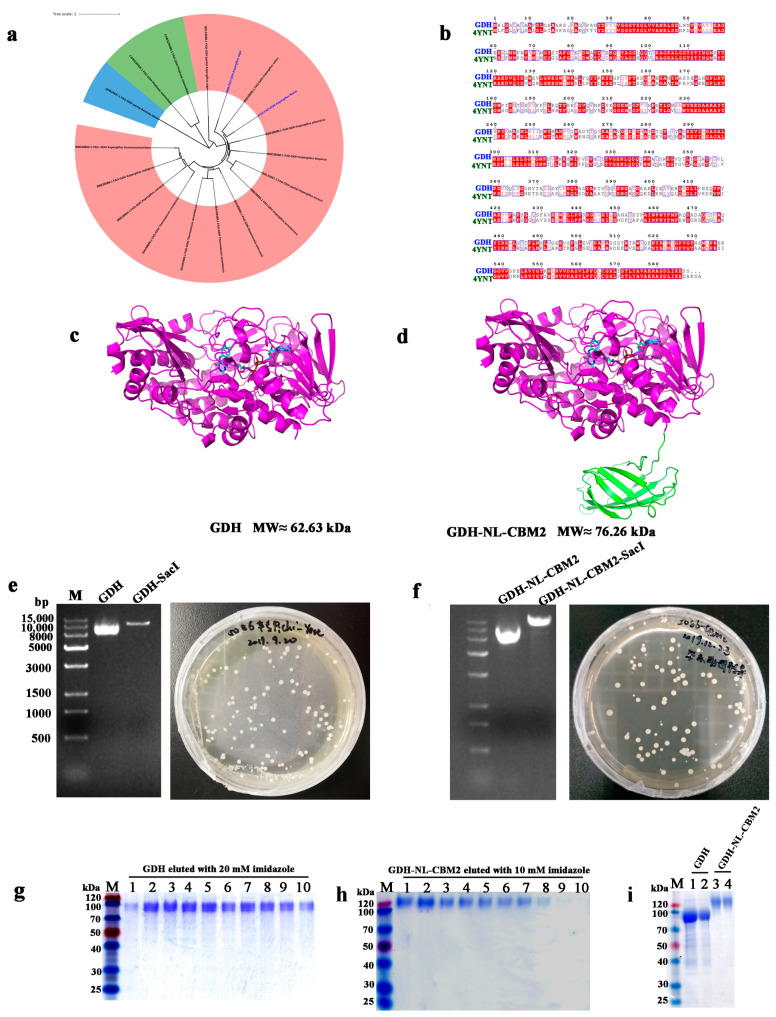
Sequence, phylogenetic, and structural analysis of FAD-GDH from *A. niger* An76 as well as SDS-PAGE analysis of heterologously expressed FAD-GDH proteins. (**a**) Phylogenetic analysis of the fFAD-GDHs retrieved from the CAZy database; (**b**) Sequence alignments of *Aspergillus flavus* FAD-GDH (AfGDH, PDB ID: 4YNT); (**c**,**d**) Predicted structures of wild-type GDH and fusion GDH (GDH-NL-CBM2), respectively; (**e**,**f**) Agarose gel electrophoresis analysis of the original and linearized plasmids of pPIC9k-GDH and pPIC9k-GDH-NL-CBM2, respectively; (**g**) Lane M: protein standard marker, Lanes 1–10: the eluting numerical sequence under the optimal imidazole elution concentrations of GDH and GDH-NL-CBM2, SDS-PAGE of GDH eluted with 20 mM imidazole; (**h**) SDS-PAGE of GDH-NL-CBM2 eluted with 10 mM imidazole; (**i**) SDS-PAGE of concentrated GDH and GDH-NL-CBM2, lanes 1 and 2, 3, and 4 were duplicate tests for GDH and GDH-NL-CBM2, respectively.

In previous studies, several novel FAD-GDH gene homologs were discovered based on genomic information analysis of FAD-GDHs [2]. In the genome of *A. niger* An76 sequenced by our lab, we found a novel FAD-GDH gene that was phylogenetically distantly related with other FAD-GDHs from *Aspergillus* species, as shown in Figure 1a, and the sequence identity was only 53.03% with that of the *Aspergillus flavus* FAD-GDH (AfGDH, PDB ID: 4YNT) [20] (Figure 1b), which has been widely used for commercial self-monitoring of blood glucose sensors. The distinct sequence characteristics of FAD-GDH from *A. niger* An76 prompted us to further study its catalytic properties.

The FAD-GDH gene from *A. niger* An76, containing a 1719 bp open reading frame, encoded a protein of 573 amino acids with a predicted molecular mass of about 62.63 kDa (Figure 1c). The initial 63 bp at the 5′end of the GDH gene, encoding a signal peptide, was deleted, and the His tag encoding gene was added at the 3′end of the GDH gene. Then, after codon-optimizing the recombinant GDH gene, it was inserted directly into the pPIC9k vector to construct the plasmid pGDH, which was compatible for wild-type GDH heterologous expression and secretion in *Pichia pastoris* GS115. The GDH-NL-CBM2 encoding gene was constructed by inserting the NL and CBM2 gene between the GDH and His tag encoding gene. The theoretical molecular weight of the NL-CBM2 (aa) was about 13.6 kDa; thus, the predicted molecular mass of GDH-NL-CBM2 was about 76.26 kDa (Figure 1d).

As shown in Figure 1e–i, the wild-type GDH and recombinant GDH-NL-CBM2 were successfully produced by *P. pastoris*, and the optimal imidazole elution concentrations of GDH and GDH-NL-CBM2 were 20 and 10 mM, respectively. The discrepancy may be attributed to the NL-CBM2 located next to the His tag in the C terminal of the fusion protein, which affected the exposure of the His tag, and thus, the binding force between the GDH-NL-CBM2 and Ni column was weaker. In addition, the molecular weight of the GDH and GDH-NL-CBM2 proteins was obviously higher than the predicted one, which may be due to the protein glycosylation in *P. pastoris*, as reported in previous study [21]. Compared to that of GDH, the molecular weight of GDH-NL-CBM2 was much larger, which suggested that the NL-CBM2 has been successfully fused with GDH.

### 2.2. Analysis of the Enzyme Activity of GDH and GDH-NL-CBM2

To verify the glucose oxidation catalytic activity of heterologously expressed GDH and GDH-NL-CBM2, the centrifuged fermentation broth of the engineered strain (induced for 5 d) was collected and used for enzyme activity assay. The results demonstrated that the enzyme activity (5485.99 U/L) of GDH was about 1.2 times that of GDH-NL-CBM2 (4531.60 U/L), indicating that both GDH and GDH-NL-CBM2 maintained the ability to oxidize glucose. Furthermore, the Michaelis–Menten constant (K_m_, V_max_) values of the purified GDH and GDH-NL-CBM2 were determined, which showed that the catalytic kinetic constant value of GDH-NL-CBM2 (K_m_ 11.8 mM, V_max_ 3.98 mM/L/min) for glucose was almost equal to that of GDH (K_m_ 12.3 mM, V_max_ 4.29 mM/L/min). Previous studies have shown that fusing large tags usually makes the protein expression more difficult and results in undesirable consequences, such as domain misfolding, low protein yield, and impaired bioactivity [22]. Accordingly, the lower enzyme activity of GDH-NL-CBM2 in fermentation supernatant may be attributed to the larger molecular weight of GDH-NL-CBM2, which hindered the expression of the enzyme and reduced enzyme yield. However, the unaffected catalytic performance of the enzyme may benefit from the sufficient space separating the binding and catalytic domains provided by the natural linker as reported previously [22].

**Figure 2:**
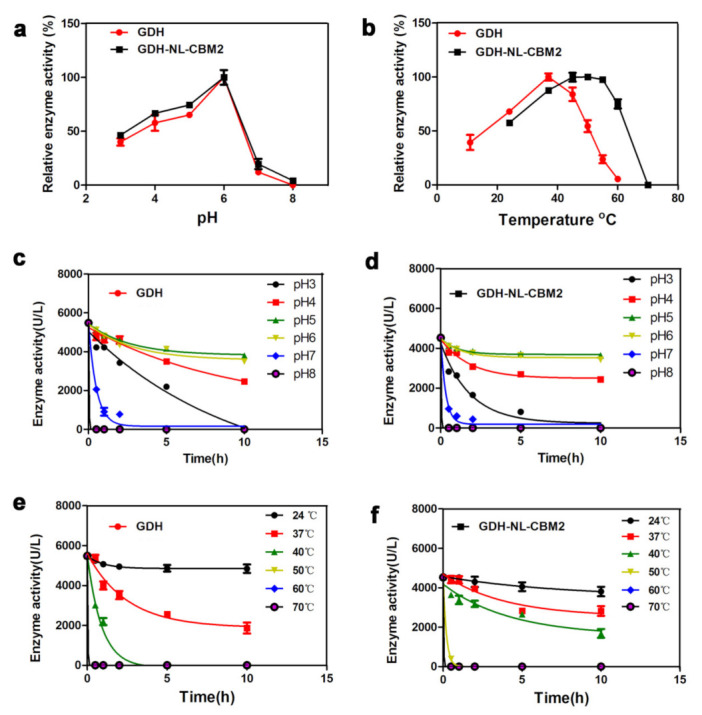
Determination of the optimal temperature and pH value as well as the pH and temperature stability for GDH and GDH-NL-CBM2. (**a**,**b**) Optimal pH and temperature of the GDH, GDH-NL-CBM2, respectively; (**c**,**d**) pH stability for the GDH and GDH-NL-CBM2, respectively; (**e**,**f**) Temperature stability for GDH and GDH-NL-CBM2, respectively.

Additionally, the effects of the pH and temperature on the activity of GDH and GDH-NL-CBM2 were examined. As shown in Figure 2, they were both highly active at pH 6.0 and exhibited good pH stability within the range of pH 5.0−6.0, but the retained activity of GDH and GDH-NL-CBM2, after incubation for 10 h at pH 5.0, was about 75% and 86%, respectively. In addition, it was notable that the enzymes displayed low activity and stability in the neutral pH 7.0 conditions. As reported in advanced studies, the surface charged residues, especially the percentage of charged amino acids (D, E, K, R), impact enzymatic conformational stability through electrostatic interactions: hydrogen bonds [23]. Therefore, the number of charged amino acids (D, E, K, R) at the surface of GDH was analyzed. The results showed that the number of acidic residues (D, E) was 1.7-fold that of alkaline residues (R, K), which suggested that the enzyme was more stable in acid conditions. The acid-stable nature of this enzyme may be related to the evolutionary adaption to gluconic acid produced by this enzyme. The optimal reaction temperature of GDH and GDH-NL-CBM2 was 37 °C and 55 °C, respectively, and after incubation for 10 h at 40 °C, 44% of the enzyme activity was retained by GDH-NL-CBM2. However, no enzyme activity was detected for GDH, indicating the higher thermal stability of GDH-NL-CBM2.

In order to determine the binding ability of the CBM2 tag in the fusion protein, the GDH and GDH-NL-CBM2 were separately mixed with nanocellulose at different temperature and pH values, and then, the protein content in centrifuged supernatants were detected. The results showed that the amount of GDH before and after the reaction did not change significantly, indicating that GDH could hardly be combined with nanocellulose. Under the same reaction conditions, GDH-NL-CBM2 could clearly bind to nanocellulose. In addition, when the reaction temperature (22, 30, 40, 50, 60, and 70 °C) increased, hardly any enzyme was detected in the supernatant at different temperature values. However, when the reaction pH value ranged from 3.0 to 8.0, almost no enzyme was detected in the supernatants at pH values from 3.0 to 5.0, but 5% enzyme was retained at pH values 6.0 to 8.0.

In previous studies, various recombinant CBM–fusion proteins have been reported, and in several cases, the stability or activity of the target proteins were enhanced after fusion with the CBM domain, but in other instances, their performance of fusion protein was no match for the native protein [14,24]. In this study, the thermal stability of GDH-NL-CBM2 was clearly improved, which may be due to the reduced flexibility of the highly flexible terminal loop after fusing with a small domain at the terminal as explained in previous studies [14,25]. In addition, the temperature and pH values had a negligible effect on the binding ability of CBM2, which was consistent with the report that the interaction of CBMs (especially, CBMs from families 2 and 3) with cellulose was so strong that they could not be easily desorbed [18]. Thus, CBM2 is a potentially suitable module to be fused with GDH and used in biosensors.

### 2.3. Morphological Characterization of the Modified Electrode Surface

**Figure 3:**
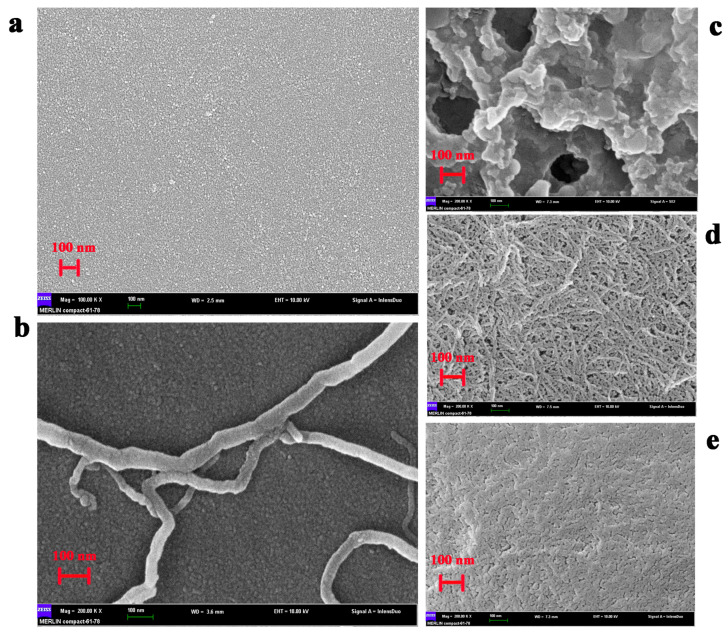
SEM images of the GCE electrode surface modified with distinct methods. (**a**) bare GCE; (**b**) S-MWNT/GCE; (**c**) GA/CS/GDH/S-MWNT/GCE; (**d**) NC/S-MWNT/GCE; (**e**) GDH-NL-CBM2/NC/S-MWNT/GCE.

The SEM analysis of the different morphological characteristics of the electrode surface modified by distinct methods revealed, as shown in Figure 3, that in the nanoscale, the surface of the bare GCE electrode was smooth (Figure 3a), but after modification with S-MWNT, several S-MWNT molecules with 40–60 nm pipe diameter were clearly observed (Figure 3b). When the S-MWNT/GCE was further modified with GDH, chitosan, and glutaraldehyde in a sandwich way, a ragged structure with large holes was observed (Figure 3c). However, when the S-MWNT/GCE was loaded with GDH-NL-CBM2 uncombined nanocellulose, the S-MWNT could still be observed, and nanocellulose with a diameter of 5–20 nm formed a thin film, but the nanofiber was obvious (Figure 3d). After reacting with GDH-NL-CBM2, the S-MWNT and nanofiber could not be observed and a denser and smoother film was obtained, which indicated that a large amount of GDH-NL-CBM2 was immobilized on the surface of the electrode.

### 2.4. Electrochemical Characterization of the Electrodes Prepared by Two Different Methods

**Figure 4:**
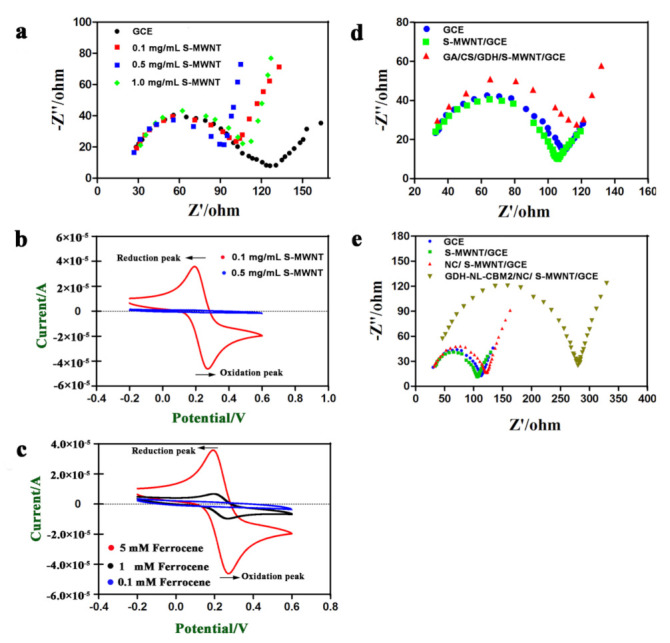
Electrochemical characterization of the electrodes prepared by two different methods**.** (**a**) Impedance spectra for bare GCE (black), 0.1 mg/mL S-MWCN/GCE (red), 0.5 mg/mL S-MWCN/GCE (blue), and 1 mg/mL S-MWCN/GCE (green) electrode; (**b**) CV of GCE modified with 0.1 mg/mL (red) and 0.5 mg/mL (blue) S-MWNT in 5 mmol/L ferrocene (Fc) solution; (**c**) CV of GCE modified with 0.1 mg/mL S-MWNT in 0.1 mmol/L (blue), 1 mmol/L (black) and 5 mmol/L (red) ferrocene solution; (**d**) Impedance spectra for bare GCE (blue), S-MWCN/GCE (green), and GA/CS/GDH/S-MWCN/GCE (red); (**e**) Impedance spectra of bare GCE (blue), S-MWCN/GCE (green), NC/S-MWNT/GCE (red), and GDH-NL-CBM2/NC/S-MWNT/GCE (brown). The measurement of EIS was performed in a 0.1 M KCl solution containing 10 mM K_3_ [Fe(CN)_6_] and K_4_ [Fe(CN)_6_] as the electrochemical probe solution. The arrows in b and c indicated the scanning direction.

As previously reported, it is an essential requirement to achieve efficient electron transfer by complete debundling of CNTs and the lower resistance of the formed CNT layer [26]. It has been shown that perfluorosulfonated polymer Nafion can solubilize single-walled and multi-walled CNTs, and the redox activity of hydrogen peroxide at CNT/Nafion-coated electrodes was dramatically enhanced [27]. Thus, a method involving Nafion-assisted dispersion was utilized in this study. In order to optimize the concentration of S-MWNT/Nafion, ESI, a powerful tool for measuring the charge transfer resistance (Rct) value at the interface between the electrode and the electrolyte and analyzing the dynamics of electron transfer [28] was used to measure the electron transfer efficiency of S-MWNT. As shown in Figure 4a, the Rct value and the straight-line angle in the low-frequency range varied for the electrodes modified with a series of concentration (0.1, 0.5, and 1 mg/mL) of S-MWNT. The electrode modified with 1 mg/mL S-MWNT had the largest Rct value, perhaps as a result of the insufficient debundling of the S-MWNT. In addition, the electrode modified with 0.5 mg/mL S-MWNT had a lower Rct value than that of the electrode modified with 0.1 mg/mL S-MWNT. However, the straight-line angle in the low-frequency range of the former was close to 90°, and it was about 45° for the latter. It has been reported that when the CNTs on the electrode interface was a monolayer, as shown in Figure 3b, and the interface is flat, the straight line in the low-frequency region tends to be 45°, and the interface is more susceptible to Faraday current [29]. Therefore, we hypothesized that the optimal performance concentration of S-MWNT may be 0.1 mg/mL, and then, the hypothesis was confirmed by the larger current response, as shown by the CV curves (Figure 4b).

Ferrocene (Fc), a highly-rich electronic system with strong aromaticity, has various advantages, such as good heat stability, high reactivity, easy structural modification, etc. In addition, it can transfer electrons from the enzyme to the electrode through a rapid redox reaction, benefiting from the good matches of the molecular dimension of the ferrocene at the enzyme active center. Thus, ferrocene and its derivatives are considered ideal redox mediators to construct mediator-based biosensors [30]. Therefore, ferrocenylmethanol was used as mediator in this study, and the concentration was optimized. As shown in Figure 4c, a couple of reversible redox peaks were observed at +0.3 V/+0.2 V, and the current of the redox peaks increased with the increase of the ferrocenylmethanol concentration. In addition, 5 mM ferrocenylmethanol, which showed an obvious electrical signal, was used.

As shown in Figure 4d,e, when a bare GCE was modified with S-MWNT, the electron transfer process was accelerated, whereas the electron transfer process was limited when the S-MWNT/GCE was further modified with enzymes and support, which corresponded with the insulating properties of the enzymes, nanocellulose and chitosan. Moreover, it was also noticeable that the Rct value of the GDH-NL-CBM2/NC/S-MWNT/GCE was much higher than that of the GA/CS/GDH/S-MWNT/GCE. This discrepancy may be explained by the denser film of the GDH-NL-CBM2/NC/S-MWNT/GCE as shown in Figure 3c,e.

### 2.5. Electrochemical Behavior of the Electrodes Prepared by Two Methods to Glucose

**Figure 5:**
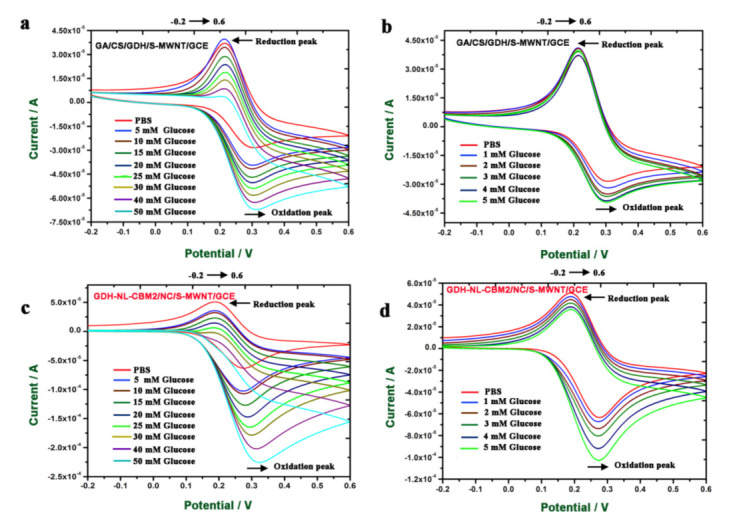
Cyclic voltammetry (CV) measurements of the GA/CS/GDH/S-MWCN/GCE and GDH-NL-CBM2/NC/S-MWNT/GCE responding to different concentrations of glucose. (**a**) CV measurements of GA/CS/GDH/S-MWCN/GCE responding to high concentrations of glucose (5, 10, 15, 20, 25, 30, 40, and 50 mM) dissolved in PBS (0.1 M citric acid/0.2 M Na_2_HPO_4_ buffer, pH 6.0, with 5 mM ferrocenylmethanol); (**b**) CV measurements of GA/CS/GDH/S-MWCN/GCE responding to low concentrations of glucose (1, 2, 3, 4, and 5 mM) dissolved in PBS (0.1 M citric acid/0.2 M Na_2_HPO_4_ buffer, pH 6.0, with 5 mM ferrocenylmethanol); (**c**) CV measurements of GDH-NL-CBM2/NC/S-MWNT/GCE responding to high concentrations of glucose (5, 10, 15, 20, 25, 30, 40, and 50 mM) dissolved in PBS (0.1 M citric acid/0.2 M Na_2_HPO_4_ buffer, pH 6.0,with 5 mM ferrocenylmethanol); (**d**) CV measurements of GDH-NL-CBM2/NC/S-MWNT/GCE responding to low concentrations of glucose (1, 2, 3, 4, and 5 mM) dissolved in PBS (0.1 M citric acid/0.2 M Na_2_HPO_4_ buffer, pH 6.0, with 5 mM ferrocenylmethanol). The arrows indicated the scanning direction.

In order to compare the electrochemical behavior of the GA/CS/GDH/S-MWNT/GCE and GDH-NL-CBM2/NC/S-MWNT/GCE, the electrochemical properties of the two electrodes in PBS (0.1 M citric acid/0.2 M Na_2_HPO_4_ buffer, pH 6.0, with 5 mM ferrocenylmethanol) buffer and different concentrations (1–50 mM) of glucose dissolved in PBS buffer were evaluated by CV at a scan rate of 50 mV s^−1^. As shown in Figure 5, when the glucose concentration was increased, the current density of the oxidation peak at a potential of +0.3 V also increased, but the reduction peak at a potential of +0.2 V decreased for both bioelectrodes. Meanwhile, the current density produced by the GDH-NL-CBM2/NC/S-MWNT/GCE was about three times that generated by GA/CS/GDH/S-MWNT/GCE, which indicated that the electron transfer of the GDH-NL-CBM2/NC/S-MWNT/GCE was more efficient. Three reactions with Fc may possibly occur, as shown in Equations (1)–(3) [31]:

Glucose + GDH (FAD)→Gluconic acid + GDH (FADH2)(1)

2Fc^+^ + GDH (FADH2)→2Fc + GDH (FAD) (2)

2Fc→2Fc^+^ + Electrode. (3)

In this reaction system, the amount of Fc in different valent states was constant; when glucose was catalyzed by FAD-GDH, a large amount of Fc was reduced, which was in accordance with the current increase by the oxidation reaction of Fc. At the same time, the amount of Fc in the oxidated state decreased, corresponding to the decrease of the reduction peak. A similar phenomenon was reported in an fc-mediated glucose oxidase biosensor study, in which a large increase in the oxidation current was observed at +0.25 V vs. Ag/AgCl in the presence of glucose and a substantial decrease in the reduction current at +0.15 V vs. Ag/AgCl [31].

**Figure 6:**
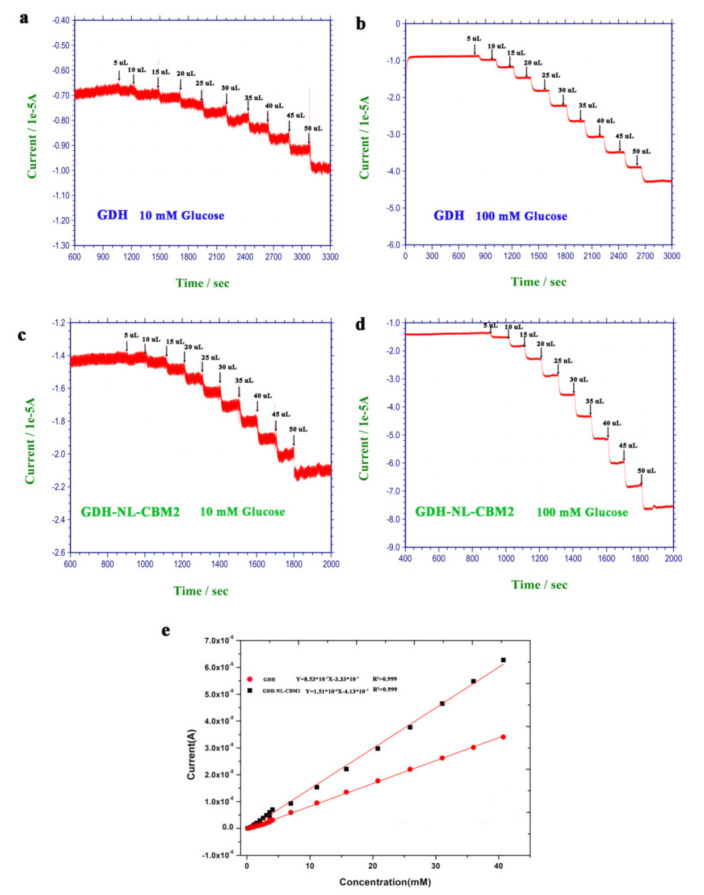
Time–current response of the GA/CS/GDH/S-MWCN/GCE and GDH-NL-CBM2/NC/S-MWNT/GCE electrodes for the sequential addition of 100 mM and 10 mM glucose at different volumes. (**a**,**b**) Addition of 10 mM and 100 mM glucose at different volumes for the GA/CS/GDH/S-MWCN/GCE, respectively; (**c**,**d**) Addition of 10 mM and 100 mM glucose at different volumes for the GDH-NL-CBM2/NC/S-MWNT/GCE, respectively; (**e**) Calibration plot for the glucose response using the data in (**a**–**d**).

In order to obtain an improved accurate sensitivity and detection limit for the two electrodes, the current–time (i-t) method was used to study the current change with successive addition of different volumes of glucose (10 or 100 mM) at +0.3 V vs. Ag/AgCl. As shown in Figure 6, the current change with the GA/CS/GDH/S-MWNT/GCE electrode responded linearly to glucose in the concentration range of 0.12–40.70 mM with a coefficient of determination R^2^ > 0.999, a linear equation of y = 8.53 × 10^−7^x − 3.33 × 10^−7^, a sensitivity of 1.2067 × 10^−2^ A/(M*cm^2^), and a detection limit of 0.081 mM (S/N = 3), and the maximum electrocatalytic response was reached ≈40 s after adding the glucose into the electrolyte solution. With respect to GDH-NL-CBM2/NC/S-MWNT/GCE, a good linear relationship was also observed between the current density and the concentration of glucose (0.12–40.70 mM, R^2^ > 0.999), with a linear equation of y = 1.51 × 10^−6^x − 4.13 × 10^−7^. However, compared with the GA/CS/GDH/S-MWNT/GCE electrode, for the GDH-NL-CBM2/NC/S-MWNT/GCE electrode, the sensitivity (2.1362 × 10^−2^ A/(M*cm^2^) was much higher, the detection limit was relatively lower (0.052 mM, S/N = 3), and the response time (≈18 s) was shorter. In addition, compared with the crosslinking method by glutaraldehyde, the affinity adsorption method exhibited clear advantages in sensitivity and response time. This discrepancy may result from the high retention rate of GDH with the correct conformation achieved by CBM binding as previously reported [13,32]. In addition, we compared the sensitivity, linear range, and response time of the biosensors developed in this study with several previously reported glucose sensors, as shown in Table 1 [26,33,34,35,36,37,38,39,40,41,42,43], which revealed that the performances of all these sensors are comparable (Table 1).

### 2.6. Specificity and Anti-Interference Studies

**Figure 7:**
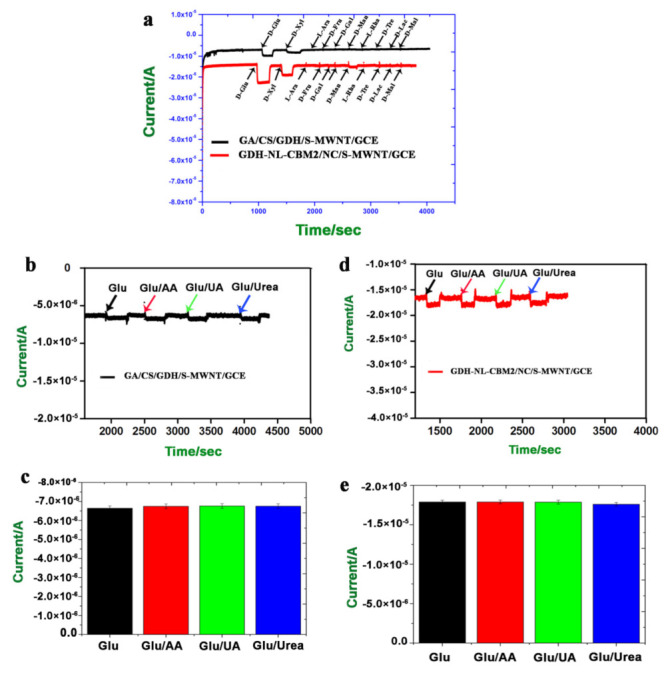
Specificity and anti-interference analysis of the GA/CS/GDH/S-MWCN/GCE and GDH-NL-CBM2/NC/S-MWNT/GCE electrodes by the time–current method. (**a**) Substrate specificity detected by adding various sugars (100 mM, 25 µL), including d-xylose, l-arabinose, d-fructose, d-galactose, d-mannose, d-rhamnose, and d-trehalose, d-lactose, and d-maltose; (**b**,**c**) Anti-interference ability of the GA/CS/GDH/S-MWCN/GCE studied by adding ascorbic acid (AA, 50 μM), uric acid (UA, 0.2 mM), and urea (2 mM) with their respective final concentrations in the human serum with 5 mM glucose; (**d**,**e**) Anti-interference ability of the GDH-NL-CBM2/NC/S-MWNT/GCE studied by adding ascorbic acid (AA, 50 μM), uric acid (UA, 0.2 mM), and urea (2 mM) with their respective final concentrations in the human serum with 5 mM glucose.

The specificity of the enzyme electrode is an important feature for specific recognition of the target substrate. In order to study the specificity and anti-interference ability of the electrodes prepared by different methods, various sugars (100 mM, 25 µL) including d-xylose, l-arabinose, d-fructose, d-galactose, d-mannose, d-rhamnose, d-trehalose, d-lactose, and d-maltose were used as substrates for evaluation. As shown in Figure 7a, besides the current response caused by d-glucose, d-xylose could also induce a reaction current for both electrodes, and the enzymatic activity for xylose was approximately 45% that for glucose, which was consistent with a previous report that xylose was a competitive substrate for FAD-GDH [2]. In addition, d-mannose was also found to be an interfering substrate for the GDH-NL-CBM2/NC/S-MWNT/GCE electrode, and the reaction activity for mannose was about 5% that for glucose. However, no current signal was detected for the reaction of the GA/CS/GDH/S-MWNT/GCE with mannose. The different response of the two electrodes to mannose may be due to the higher current density generated by the GDH-NL-CBM2/NC/S-MWNT/GCE.

The anti-interference ability of the two electrodes was studied by adding ascorbic acid (AA, 50 μM), uric acid (UA, 0.2 mM), and urea (2 mM) to their respective final concentration in the human serum with 5 mM glucose and used as assay substrates. The results showed that negligible changes in the current signal (AA 1.52%, UA 1.78%, and urea 1.63%) occurred with the GA/CS/GDH/S-MWNT/GCE electrode (Figure 7b,c). In addition, more negligible changes (AA 0.01%, UA 0.15%, and urea 1.66%) were detected with the GDH-NL-CBM2/NC/S-MWNT/GCE electrode (Figure 7d,e). These results indicated that the presence of interfering substances in the serum hardly affected the process of glucose detection.

### 2.7. Stability and Reproducibility of the Electrodes

**Figure 8:**
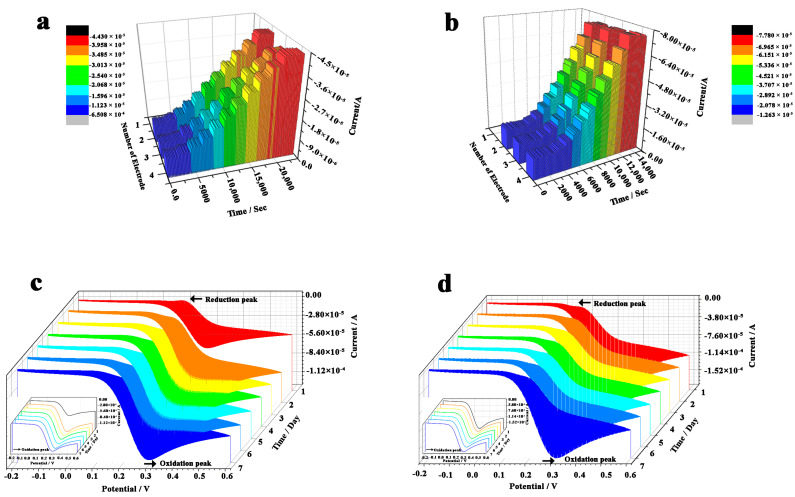
Analysis of the reproducibility and stability of the GA/CS/GDH/S-MWCN/GCE and GDH-NL-CBM2/NC/S-MWNT/GCE electrodes. (**a**,**b**) The reproducibility of three independent electrodes prepared with the GA/CS/GDH/S-MWCN/GCE and GDH-NL-CBM2/NC/S-MWNT/GCE, respectively, and the current changes with successive addition of different volumes of glucose (100 mM) at +0.3 V vs. Ag/AgCl were measured by the amperometric current–time curve method; (**c**,**d**) The corresponding stability of the GA/CS/GDH/S-MWCN/GCE and GDH-NL-CBM2/NC/S-MWNT/GCE electrodes evaluated by CV in 20 mM glucose in a week. The insert maps in c and d presented the oxidation reaction. The arrows in c and d indicated the scanning direction.

The reproducibility of GA/CS/GDH/S-MWNT/GCE and GDH-NL-CBM2/NC/S-MWNT/GCE electrodes was investigated using three independent electrodes prepared for each kind of modified electrodes. The current changes with the successive addition of different volumes of glucose (100 mM) at +0.3 V vs. Ag/AgCl were measured by the amperometric i-t method. The results revealed that higher reproducibility was obtained with the GDH-NL-CBM2/NC/S-MWNT/GCE (relative standard deviation (RSD) < 5%) compared with the GA/CS/GDH/S-MWNT/GCE (RSD < 10%) (Figure 8a,b), but the two electrodes prepared in this study exhibited a comparable performance to that of other electrodes previously reported (Table 1).

In addition, the stability was evaluated by CV in different concentrations of glucose (5, 10, 15, 20, 25, 30, 40, and 50 mM; in PBS (0.1 M citric acid/0.2 M Na_2_HPO_4_ buffer, pH 6.0)) in a week, and the redox peak currents induced by 20 mM glucose in seven successive days were compared, as shown in Figure 8c,d. The results showed that the current response of the oxidation peak remained almost at 98.6% of the initial response for the GDH-NL-CBM2/NC/S-MWNT/GCE, while for the GA/CS/GDH/S-MWNT/GCE electrode, a considerable increase (83%) of the current density was observed at day 2 compared with that at day 1, and in the six successive days, the change of its current response was negligible, and approximately 98% of its current response at day 2 was retained. The significant current increase with the GA/CS/GDH/S-MWNT/GCE at day 2 suggested that the electrode modified in a sandwich way required activation for 24 h before normal detection. These results further confirmed that the GDH-NL-CBM2/NC/S-MWNT/GCE electrode has better stability and reproducibility.

### 2.8. Determination of Glucose in Rat Serum Samples and Glucose Drinks

In order to explore the possible application of the developed biosensor, the GA/CS/GDH/S-MWNT/GCE and GDH-NL-CBM2/NC/S-MWNT/GCE electrodes were used to measure the glucose concentration in the serum of pregnant rats and glucose drinks, measuring each sample in triplicate. As shown in Table 2, the RSD of each sample measured by the GA/CS/GDH/S-MWNT/GCE and GDH-NL-CBM2/NC/S-MWNT/GCE electrodes was approximately 2.9 and 1.8, respectively, which was comparable to that measured by SBA glucose biosensor (≈1.9%) and reducing sugar analyzer (≈1.6%), indicating the good reproducibility and measurement accuracy of the electrodes fabricated in this study. In addition, when glucose drinks with standard glucose concentration was analyzed, the concentration of glucose obtained by the GDH-NL-CBM2/NC/S-MWNT/GCE was the closest to the actual value. In addition, with respect to the glucose concentration in the serum of pregnant rats, the results detected by GDH electrodes were comparable with that obtained by the reducing sugar analyzer and the SBA glucose biosensor. The results showed that the electrodes developed in this study have a great potential for practical application in the determination of glucose in actual samples.

## 3. Materials and Methods

### 3.1. Sequencing and Phylogenetic Analysis

All protein sequences of fFAD-GDHs were retrieved from the Carbohydrate-Active enZYmes database (CAZy, http://www.cazy.org/, accessed on 6 July 2020). The CLUSTAL algorithm implemented in the MEGA5 software was used to perform multiple sequence alignments. A neighbor-joining phylogenetic tree was constructed with a bootstrap test based on 18 members with definite annotation, and further optimized by iTOL (https://itol.embl.de/, accessed on day 10 July 2020). Sequence alignment produced was plotted using ESPript 2.2 (http://imed.med.ucm.es/ESPript/ESPript/index.php, accessed on day 10 July 2020). The predicted structure of the FAD-GDH from *A. niger* An76 was generated by SWISS-MODEL (https://swissmodel.expasy.org/, accessed on day 12 July 2020) and displayed by the PyMOL software (1.7, DeLano Scientific, San Carlos, CA, USA).

### 3.2. Materials and Chemical Reagents

Yeast extract and tryptone were bought from Oxoid Ltd. (Basingstoke, UK); (+)-biotin, disodium hydrogen phosphate (Na_2_HPO_4_), imidazole, tris(hydroxymethyl)aminomethane (Tris), glycine, acrylamide, bis-acrylamide, ammonium persulfate, N,N,N′,N′-tetramethylethylenediamine (TEMED), and Coomassie brilliant blue protein assay kit were purchased from Sangon Biotech (Shanghai, China); methanol, glycerol, glucose, ethanol, and citric acid were supplied by Sinopharm (Beijing, China); yeast nitrogen base (YNB) without amino acids and ammonium sulfate, phenazine methosulfate (PMS), 2,6-dichlorophenolindophenol (DCIP), Nafion, and chitosan were purchased from Sigma–Aldrich Corp. (St. Louis, MO, USA); nanocellulose (10–200 nm) was purchased from North Century Cellulose Material Co., Ltd. (Suining, China); restriction enzymes (Not I, Sac I and EcoR I), and the Capturem™ His-Tagged Purification Miniprep Kit were purchased from Takara (Dalian, China); multi-walled carbon nanotubes (S-MWNT-4060) were purchased from Shenzhen Nanotech Port Co., Ltd. (Shenzhen, China). All other chemicals were reagent grade.

*Escherichia coli* DH5α strain and the plasmid extraction kit were purchased from Vazyme Biotech Co., Ltd. (Nanjing, China); the Cycle Pure Kit was obtained from Omega Bio-Tek (Norcross, GA, USA); Pichia pastoris GS115, used as protein expression host, was maintained by our lab, the pPIC9k vector (Invitrogen, Carlsbad, CA, USA) was used as an expression vector.

### 3.3. Construction of Recombinant Plasmid

The corresponding gene sequences of GDH and CBM2 from *A. niger* [44] and *Thermobifida fusca* [45], respectively, were codon optimized using the Codon OptimWiz software (https://www.genewiz.com.cn/, accessed on day 2 August 2020), and the optimized gene sequence of the GDH was cloned into the restriction sites of Not I and EcoR I in pPIC9k, which is an efficient plasmid for the expression of recombinant exogenous genes in *P. pastoris*. To construct the C-terminal CBM2 fusion GDH, a natural linker (NL) between CBM2 and the catalytic domain of endo-β-xylanase (EM_PRO:Z81013.1) was used as the linker in the fusion GDH. The gene sequences, including those of the codon optimized GDH, CBM2, and NL, were synthesized using the DNA 2.0 expression system (Atum, Newark, CA, USA) and cloned into the restriction sites of Not I and EcoR I in pPIC9k to obtain the expression vectors of the corresponding C-terminal CBM fusion protein (GDH-NL-CBM2).

### 3.4. Heterologous Expression of GDH and GDH-NL-CBM2 in Pichia pastoris

The plasmids for wild-type GDH and GDH-NL-CBM2 were separately transformed into *P. pastoris* GS115 according to the manufacturer’s instructions (Invitrogen, Pichia Expression version G). MD medium (13.4 g/L YNB, 10 g/L glucose, 0.4 mg/L biotin) was used to select *P. pastoris* transformants. Several single colonies were inoculated in YPD medium (10 g/L yeast extract, 20 g/L tryptone, 10 g/L glucose) and cultured overnight at 30 °C, with shaking at 250 rpm. Then, the cultures were transferred to BMGY medium (13.4 g/L YNB, 11.8 g/L KH_2_PO_4_, 2.9 g/L K_2_HPO_4_, 10 g/L yeast extract, 10 g/L tryptone, 0.4 mg/L biotin, 1% (*v*/*v*) glycerol) and grown at 30 °C, with shaking at 250 rpm until OD600 = 2–6. Then, the cells were harvested by centrifugation, inoculated in BMMY medium (13.4 g/L YNB, 11.8 g/L KH_2_PO_4_, 2.9 g/L K_2_HPO_4_, 10 g/L yeast extract, 10 g/L tryptone, 0.4 mg/L biotin, 1% (*v*/*v*) methanol), and cultured at 30 °C and shaking at 250 rpm for 5 days, adding 1% methanol every 24 h to induce recombinant protein expression.

### 3.5. Purification of GDH and GDH-NL-CBM2

Culture supernatants containing the heterologously expressed enzymes were collected by centrifugation at 10,000× *g* for 10 min and filtered through a 0.22 μm polyethersulfone (PES) filter membrane (Millipore GmbH, Eschborn, Germany). Then, the enzymes in the supernatants were purified using His60 Ni Superflow Resin & Gravity Columns (Takara), according to the manufacturer’s instructions and eluted with a series of imidazole concentration gradients (5, 10, 20, 30, 40, 50, 60, 100, and 250 mM). The protein solution was concentrated using 30-kDa cutoff ultrafiltration tubes (Pall Corp., Port Washington, NY, USA) and transferred to citric acid/Na_2_HPO_4_ (pH 6.0). The protein concentration was measured using a Coomassie brilliant blue protein assay kit (Sangon Biotech) and identified by sodium dodecyl sulfate-polyacrylamide gel electrophoresis (SDS-PAGE).

### 3.6. Determination of Optimum Temperature and pH Value

The method used to determine the GDH activity was modified from a previous report [28]. Briefly, the reaction mixture, consisting of 1.75 mL phosphate buffer (0.1 M, pH 6.0), 0.1 mL D-glucose (2 M), 50 μL PMS (24 mM), and 50 μL DCIP (2.4 mM) was mixed thoroughly and preheated at 37 °C for 5 min. Then, 50 μL of appropriately diluted enzyme solution was added to the reaction mixture and reacted at 37 °C for 3 min. Finally, the enzyme activity was determined by measuring the molar absorption coefficient of oxidized DCIP at 600 nm (16.3 mM^−1^·cm^−1^). The heat-inactivated enzyme solution was used as a blank control, and a series of concentrations of DCIP were used to prepare a standard curve. In addition, different citric acid/Na_2_HPO_4_ buffer (pH 3.0–8.0) and temperatures (22–70 °C) were used to determine the optimal reaction pH and temperature for GDH, respectively. In addition, the enzyme was added to citric acid/Na_2_HPO_4_ buffer (pH 3.0–8.0) for different time periods (0.5, 1, 2, 5, and 10 h) to determine the pH stability of GDH. The enzyme in citric acid/Na_2_HPO_4_ buffer (pH 5.0) was incubated at different temperatures (24, 37, 40, 50, 60, and 70 °C) for different time intervals (0.5, 1, 2, 5, and 10 h) to determine the temperature stability of GDH. Kinetic analyses of the catalytic reactions were performed under standard GDH activity determination conditions using various concentrations of glucose. The Michaelis–Menten constant (K_m_, V_max_) were non-linearly fitted based on the Michaelis–Menten equation using Prism 5.0 (GraphPad Software Inc., San Diego, CA, USA).

In order to determine the optimal reaction temperature and pH value between CBM2 in GDH-NL-CBM2 and cellulose, 0.1 mL GDH-NL-CBM2 (0.5 mg/mL) was reacted with 0.9 mL nanocellulose (1.5 mg/mL) at different temperatures (25–70 °C) or pH values (pH 3.0–8.0) for 10 min. Then, the reaction mixture was centrifuged at 13,000 rpm for 3 min, the supernatant was collected, and the protein content of the supernatant was determined.

### 3.7. Preparation of GA/CS/GDH/S-MWNT/GCE and GDH-NL-CBM2/NC/S-MWNT/GCE

The method for preparing the GDH electrode was modified from a previous study [46]; the bare glassy carbon electrode (GCE) was polished with 0.05 μm alumina powder for 5 min and ultrasonically cleaned in absolute ethanol and then ultrapure water. The cleaned electrode was dried with N_2_. The S-MWNT-4060 (0.1 mg) was added to a 0.1% Nafion aqueous solution (1 mL) and sonicated for 2 h until a black homogeneous suspension (0.1 mg/mL) was obtained. Then, a 7 μL aliquot of the obtained S-MWNT/GCE suspension was dropped on the surface of the freshly polished GCE, and the electrode was dried in air. Subsequently, 14 μL (10 U, 0.028 mg) of the purified GDH solution was transferred to the surface of S-MWNT/GCE and dried in air. Then, at room temperature, 5 μL of chitosan solution (CS 0.5%, pH 5.0) was added on the surface of the GDH/S-MWNT/GCE electrode. After drying, 2 μL of a glutaraldehyde solution (GA, 25%) was dropped on the surface of the CS/GDH/S-MWNT/GCE and allowed to dry in air. Finally, the GA/CS/GDH/S-MWNT/GCE electrode was rinsed with a phosphate-buffered saline (PBS) solution (0.1 M citric acid/0.2 M Na_2_HPO_4_ buffer, pH 6.0) and stored at 4 °C for subsequent use.

To prepare a GDH-NL-CBM2 electrode, nanocellulose (NC, 500 μL, 2 mg/mL) was added to 0.5% Nafion aqueous solution (5 mL) and sonicated for 30 min to obtain a homogeneous nanocellulose solution. Then, 50 μL of this homogeneous solution was mixed with purified GDH-NL-CBM2 (10 U, 0.035 mg) and allowed to stand for 10 min. This mixture was centrifuged at 13,000 rpm for 3 min, and the supernatant without enzyme activity was completely removed. The precipitate (10 U, 0.035 mg) was redissolved in 50 μL PBS solution and mixed with 0.5% Nafion aqueous solution (7 μL) to ultimately obtain a solution of nanocellulose combined with GDH-NL-CBM2. Then, 7 μL of nanocellulose combined with the GDH-NL-CBM2 solution (1.23 U, 0.0042 mg) was dropped onto the surface of the S-MWNT-4060 modified GCE and allowed to dry in air. Finally, the GDH-NL-CBM2/NC/S-MWNT/GCE electrode was rinsed with a PBS solution and stored at 4 °C for subsequent use.

### 3.8. Electrochemical Measurements

All electrochemical experiments in this study were performed using a CHI760D electrochemistry workstation (Shanghai Chenhua Apparatus Corporation, Shanghai, China) to characterize, optimize, and calibrate the biosensor. A conventional three-electrode system was used, including a platinum counter electrode, an Ag/AgCl (3 M KCl) reference electrode, and either a bare or modified GCE (3 mm diameter) working electrode.

Various techniques, including cyclic voltammetry (CV), amperometric i-t method, and electrochemical impedance spectroscopy (EIS) analysis were used to characterize the performance of the enzyme-modified electrode. At least three parallel experiments were conducted for electrochemical measurements, and the average values were calculated and used.

The CV experiment was carried out in a static solution at a scan rate of 50 mV s^−1^ in an electrochemical cell containing a series of glucose concentrations in 5.0 mL 0.1 M citric acid/0.2 M Na_2_HPO_4_ buffer PBS (pH 6.0) with 5 mM ferrocenylmethanol. The amperometric i-t experiment was performed in an electrochemical cell manufactured in our lab, and different volumes of glucose solution (10 mM or 100 mM) were continuously added to the electrochemical cell when the working voltage was at the oxidation peak voltage of the ferrocene (+0.24 V vs. Ag/AgCl)).

The EIS analysis was performed using 0.1 M KCl solution containing 10 mM K_3_[Fe(CN)_6_] and K_4_[Fe(CN)_6_] as the electrochemical probe solution. In the frequency range from 5 × 10^−3^ to 1 × 10^5^ Hz, a sinusoidal potential modulation of ±10 mV amplitude was superimposed on the formal potential measured from the ferro-/ferric-cyanide redox couple.

The morphology of the bare or modified electrode was characterized by scanning electron microscopy (SEM) using a JSM-7800F field emission scanning electron microscope (JEOL Ltd., Tokyo, Japan).

### 3.9. Detection of Glucose in Rat Serum and Drink Samples

Serum samples of pregnant rats were provided by the Engineering Research Center of Zebrafish Models for Human Diseases and Drug Screening of Shandong Province (Jinan, China). Glucose drinks with standard concentration (50%, *w*/*v*; ≈2777.8 mM) was purchased from Jilin Tianrui Biotechnology Co., Ltd. (Jilin, China). In the three-electrode system, the GA/CS/GDH/S-MWNT/GCE and GDH-NL-CBM2/NC/S-MWNT/GCE electrodes were used as working electrode, respectively. In order to validate and compare the values measured by the two electrodes, the serum and drink samples were analyzed using a reducing sugar analyzer and SBA-glucose biosensor based on glucose oxidase.

## 4. Conclusions

This study found a novel FAD-GDH gene phylogenetically distantly related with other FAD-GDHs from *Aspergillus* species, and with low sequence identity to that of commercially used *Aspergillus flavus* FAD-GDH (AfGDH, PDB ID: 4YNT). The novel wild-type FAD-GDH and its fusion enzyme (GDH-NL-CBM2) with a CBM2 tag and a natural linker were successfully heterogeneously expressed in *P. pastoris*. Compared with the wild-type GDH, the yield of GDH-NL-CBM2 decreased slightly, but its catalytic performance was highly maintained. In addition, the wild-type GDH and GDH-NL-CBM2 were immobilized on electrode in a random and oriented way, respectively. The oriented immobilized GDH exhibited much better sensing performance, such as higher sensitivity, anti-interference ability, and better reproducibility and stability. This study is helpful not only for the construction of fusion oxidoreductases, but also for the development of a fast, sensitive, stable and anti-interference amperometric biosensor.

## Figures and Tables

**Table 1:** Comparison of the performance parameters of the glucose biosensors based on GOx and FAD-GDH.

Modified Electrode	Linear Range (mM)	Sensitivity (A/(M*cm^2^))	Detection Limit (µM)	Response Time (s)	RSD	Ref
GOx/PdNPs/CS-GR ^[a]^	0.001–1.0	3.12 × 10^−2^	0.2	≈10	5.1%	[33]
GOx/Pd NPAs/GCE ^[b]^	0.04–22	--	6.1	--	5.1%	[34]
GOx–GQD/CCE ^[c]^	0.005–1.27	8.5 × 10^−2^	1.73	~3	5%	[35]
GOx/rGO/GCE ^[d]^	0.1–27	1.85 × 10^−3^	--	<5	4.9%	[36]
GOx/AuNPs-EGr/SPCE ^[e]^	0.05–1.6	2.55 × 10^−1^	2.5	--	3.8%	[37]
TNT-GNP/[Demin]Br/ Nafion/GOx/GCE ^[f]^	0.01–1.2	5.1 × 10^−3^	--	--	--	[38]
GOx/rGO-Zn-Ag/GCE	0.1–12.0	--	10.6	--	6.7%	[39]
GDH-GBP/Au ^[g]^	3–30	1.33 × 10^−3^	3410	5–30	<10%	[40]
PPF/GDH/SWCNT-SC/PPF/Au ^[h]^	0.05–3.2	1.10 × 10^−1^	0.83	7	--	[26]
rGO/PTZ-O/GDH/GCE ^[i]^	0.5–12	4.2 × 10^−2^	--	--	--	[41]
CS/GDH/MWCNT ^[j]^	0.07–0.62	2.05 × 10^−2^	4.2	60	--	[42]
DM/GDH/GCE ^[k]^	5–30	5.3 × 10^−3^	10	200	5%	[43]
GA/CS/GDH/S-MWNT/GCE	0.12–40.7	1.2067 × 10^−2^	81	~40	<10%	This work
Nafion/GDH-NL-CBM2/S-MWNT/GCE	0.12–40.7	2.1362 × 10^−2^	51	~18	<5%	This work

[a] GOx glucose oxidase; Pd NPAs Pd nanoparticles; CS-GR chitosan–graphene; [b] Pd NPAs Pd nanoparticle assemblies; [c] GQD graphene quantum dots; CCE carbon ceramic electrode; [d] rGO Reduced graphene oxide; [e] AuNPs gold nanoparticles; EGr electroactivated graphite; SPCE screen-printed carbon electrode; [f] TNT titanate nanotubes; GNP gold nanoparticle; [g] GBP gold binding peptide; [h] PPF plasma-polymerized film; SC sodium cholate; SWCNTs single-walled carbon nanotubes; [i] PTZ-O phenothiazine–toluidine blue O; [j] MWCNT multi-walled carbon nanotubes; [k] DM dialysis membrane.

**Table 2:** Measurement of glucose in rat serum samples and glucose drinks using electrodes developed in this study and conventional methods.

Concentrations of Glucose (mM) (RSD, *n* = 3)								
Samples	SBA Glucose Biosensor		Reducing Sugar Analyzer		GA/CS/GDH/S-MWNT/GCE		GDH-NL-CBM2/NC/S-MWNT/GCE	
	Average Value	RSD /%	Average Value	RSD /%	Average Value	RSD /%	Average Value	RSD /%
Glucose drink (≈2777.8 mM)	2882.7	1.51	2793.6	1.5	2917.3	2.64	2832.1	1.59
Pregnant mouse serum	9.5	1.95	9.35	1.62	9.14	2.93	9.29	1.85

## Data Availability

No new data were created or analyzed in this study. Data sharing is not applicable to this article.
